# Atypical Craniosynostosis with Torticollis and Neurological Symptoms: A Rhombencephalosynapsis Sequence

**DOI:** 10.1155/2009/919463

**Published:** 2009-12-16

**Authors:** Virve Koljonen, Junnu Leikola, Leena Valanne, Jyri Hukki

**Affiliations:** ^1^Department of Plastic Surgery, Helsinki University Hospital, 00029 Helsinki, Finland; ^2^Helsinki Medical Imaging Centre, Department of Radiology, Helsinki University Hospital, 00029 Helsinki, Finland; ^3^Cleft Lip and Palate and Craniofacial Centre, Department of Plastic Surgery, Helsinki University Hospital, 00029 Helsinki, Finland

## Abstract

*Purpose*. We describe a case of 3-year-old girl with rhombencephalosynapsis, a rare cerebellar anomaly. *Patient*. A 3-year-old girl was admitted to our hospital due to congenital torticollis and asymmetry of face, skull and trunk. Craniosynostosis was suspected due to abnormal head shape. 3D-CT revealed closure of the sagittal suture without scaphocephalic skull. Due to atypical craniosynostosis with neurological symptoms, brain-MRI was performed revealing rhombencephalosynapsis. *Results*. Our patient presented with atypical craniosynostosis and balance problems, not typical for scaphocephaly. Operative treatment for craniosynotosis was not carried out because the cause of the problems was the cerebellum instead of the brain. *Conclusions*. Therefore, we conclude that patients with atypical craniosynostosis should be examined with brain-MRI to exclude the intracranial malformations, which 3D-CT does not reveal. Without brain-MRI, decision (not) to perform surgery could have been different.

## 1. Introduction

Rhombencephalosynapsis (RES) is a rare malformation of the posterior fossa, characterised by vermian agenesis or severe hypogenesis, fusion of the cerebellar hemispheres, and closely apposed or fused dentate nuclei. Various supratentorial anomalies are occasionally seen [1–3]. Only about 40 cases are described in literature since 1916 when was first described by Obersteiner in autopsy [4]. The diagnosis is based on imaging findings due to ambiguous clinical presentation. Magnetic resonance imaging (MRI) is the only reliable method for diagnosing RES [5, 6].

## 2. Case Report

**Figure 1:**
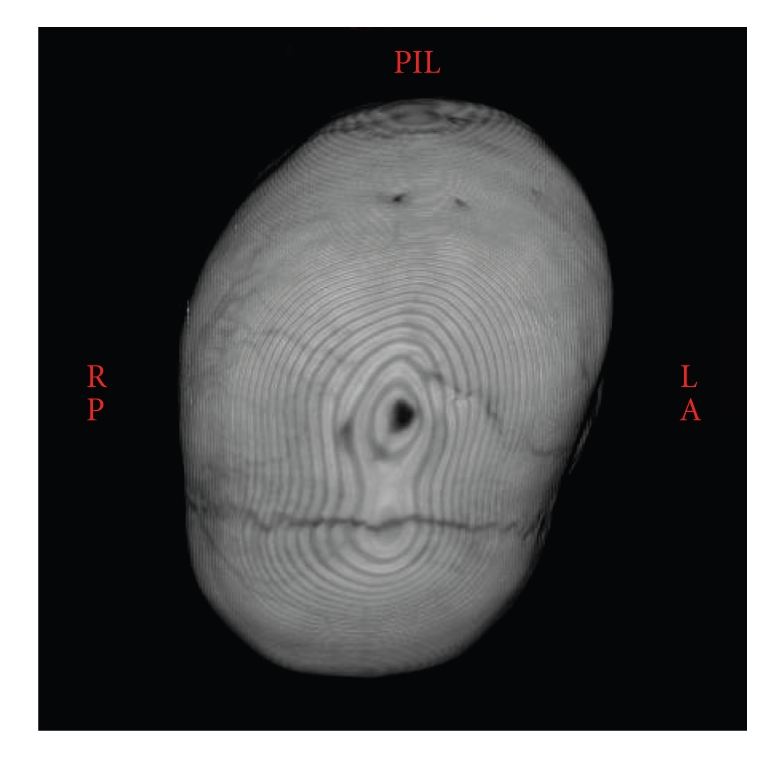
Three-dimensional CT showing the synostotic sagittal suture with a posteriorly twisted skull.

A 3-year-old girl was admitted to our hospital due to congenital torticollis and asymmetry of face, skull, and trunk. She was delivered normally after normal pregnancy. Parental history was also normal. At early age, she developed balance problems and deterioration in fine-motor skills. Initially, craniosynostosis was suspected due to abnormal head shape. Due to progressive asymmetry of skull and face a 3D-CT was performed, revealing a closure of the sagittal suture without typical scaphocephalic skull form, with no abnormalities at lambdoid sutures, Figure 1. 

In the clinical examination, narrow, posteriorly crooked skull with facial asymmetry and left-sided hypoplasia and hypotony in the truncus and extremities was noted. Facial expressions were hypomimic. There was a distinct deviation of the facial axis in an anticlockwise direction. A slight prominence in the processus mastoideus region was observed, indicating a tension in the muscle insertion area. In the neck musculature, no tension or spasms in the left sternocleidomastoid muscle or in the trapezius muscle were observed. The passive neck movement ranges were free. The spine was radiologically examined as whole, and no cervical anomalies including the anomalies of the atlas, dens, and the junction were found. The ultrasound image of the neck musculature revealed that the left sternocleidomastoid muscle was 2 mm thinner compared with the right-sided muscle. Intellectual status in repeated examinations was normal for her age. No signs of the trochlear palsy as a cause of the torticollis were found in the ophthalmologic or neurologic examinations.

**Figure 2:**
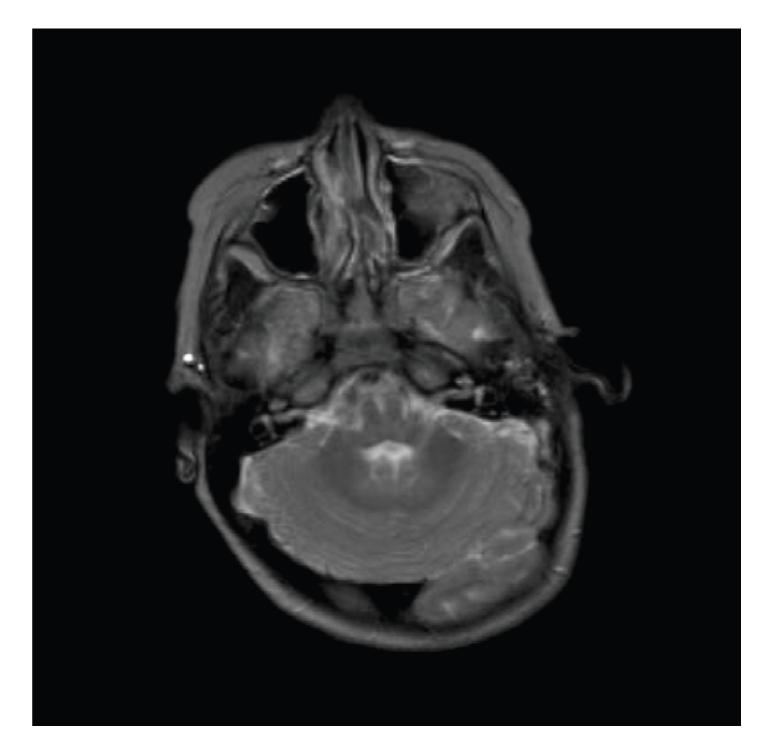
Magnetic resonance axial T2-weighted imaging in a patient with rhombencephalosynapsis shows fusion of the vermis and cerebellar hemispheres.

Due to atypical synostosis and persistent torticollis with neurological signs, a brain-MRI was performed. This revealed partial fusion of the cerebellar hemispheres with no other cerebellar anomalies, a condition that is referred to as rhombencephalosynapsis (RES); see Figure 2. 

Our patient presented with balance problems associated with torticollis with no muscular spasms and free passive ranges of the movements. The preferred operative treatment for torticollis is dissection of the adjacent muscles. However, in our patient's case, we think that this would not have solved the problem, because the torticollis was not muscle bound. We suspect that the torticollar position of the neck is sequence to the malformed cerebellum. Further, no operative treatment typical for craniosynotosis was not carried out because the cause of the problems was cerebellum instead of the brain.

## 3. Discussion

We reported a case of atypical craniosynostosis with congenital torticollis and balance problems in a three-year-old girl. The 3D-CT revealed a premature closure of the sagittal suture with no signs of associated skull deformities. Succeeding brain-MRI revealed a rare malformation rhombencephalosynapsis (RES). 

RES is a rare posterior fossa malformation characterised by absent or hypoplastic vermis and midline fusion of the cerebellar hemispheres. Associated midline anomalies include fusion of the tectum, fused thalami, callosal dysgenesis, and absent septum pellucidum [7–10].

Because of the ambiguous and variable clinical manifestation, it was usually, at least in the past, diagnosed in the autopsy [4]. The first cases reported in living patients are from the year 1991 by Truwit et al. and Savolainen et al. [1, 11]. Nowadays, due to technical advancement in the field of neuroimaging, this disease has attracted the attention of researches in living patients [1, 3, 12]. MRI has also revealed several adult patients with RES [9, 13, 14]. 

The clinical presentation is variable, ranging from early death to variable degrees of cerebellar dysfunction and developmental delay. Most of the patients reported, suffer from mental retardation, hydrocephalus, and truncal ataxia [7, 8, 15]. On the other hand, some authors have reported RES patients with compatible cognitive functions [8, 16].

Other organs, than the brain, seem to be involved in RES, the most common being musculoskeletal system. These anomalies include upper extremity anomalies, hypoplasia of radius and absence of thumb with multiple cervical and thoracic vertebrae defects [17], phalangeal hypolasia, and poly- and syndactylia [18, 19].

Abnormal facial structures have been described by Sergi et al. in a 23-week-old aborted fetus with unilateral atresia of the external ear, ocular hypertelorism, and a broad nasal bridge [19]. 

In living patients, hypertelorims, low-set ears, and arched palate have been described [1, 2]. Of the skull deformities, craniosynostosis, occipital bone deformity has been described earlier [1]. Despite or rather due to several anomalies and deformities, no known clinical syndrome has been associated with this disorder.

Of the chromosomal aberrations only Truwit demonstrated deletion of 2q in their patient population [1]. Romanengo proposed autosomal recessive inheritance [20]. They based his observation on consanguineous parents of one RES patient. De Jong and Kirby presented three cases of defects of blastogenesis and predominantly midline defects [21]. In two of his patients one presented with RES and severe hydrocephalus, conotruncal defect, ambiguous genitalia, and unilateral preaxial polydactyly. The other patient had small cerebellum associated with hydrocephalus, cleft lip and palate, and a large sacrococcygeal teratoma. 

It seems that RES can be associated with almost any kind of anomaly or malformation. The most severe forms of anomalies are presented with spontaneously aborted fetuses or with patients who have died early in the childhood. In the past, this may have easily led to the conclusion that RES is a lethal anomaly, especially when the diagnosis was set in the autopsy. MRI has increased our knowledge about this condition, and it seems that patients with RES can survive way beyond teenage years.

Previously, Danon et al. described a patient with similar clinical symptoms with our present case [3]. These two cases share the features of balance problems and torticollis. Apart from our patient, Danon reported hydrocephalus and intellectual delay, which was not eminent in our patient. Delays in motor skills, problems with balance, and controlling truncal movements and truncal ataxia have been described to associate with RES [8, 18, 22]; all these actions are controlled by cerebellum which is affected by RES. 

In our patient's case operative treatment typical for craniosynotosis or torticollis was not carried out because the cause of the problems was of malformed cerebellar origin instead of the brain or muscles. Therefore, we conclude that patients with atypical craniosynostosis should be examined also with brain-MRI to exclude the intracranial, malformations of the brain, which the 3D-CT does not reveal. Without good imaging with brain- MRI, our decision not to perform surgery could probably have been different. Moreover, we suggest that condition with the above mentioned neurological symptoms together with RES should be called “rhombencephalosynapsis sequence.”
